# Associations between neutrophil percentage to albumin ratio and rheumatoid arthritis versus osteoarthritis: a comprehensive analysis utilizing the NHANES database

**DOI:** 10.3389/fimmu.2025.1436311

**Published:** 2025-01-23

**Authors:** Wenquan Ding, Rui La, Shenghao Wang, Zhiyuan He, Dinghua Jiang, Zhigang Zhang, Hao Ni, Wu Xu, Lixin Huang, Qian Wu

**Affiliations:** ^1^ Department of Orthopedic Surgery, Orthopedic Institute, The First Affiliated Hospital of Soochow University, Soochow, Jiangsu, China; ^2^ Department of Pathology, The First Affiliated Hospital of Soochow University, Soochow, Jiangsu, China; ^3^ Research Institute of Clinical Medicine, Jeonbuk National University Medical School, Jeonju, Republic of Korea

**Keywords:** rheumatoid arthritis, osteoarthritis, neutrophil percentage to albumin ratio, inflammation, National Health and Nutrition Examination Survey, cross-sectional study

## Abstract

**Objectives:**

The association between the neutrophil percentage to albumin ratio (NPAR) and the risk of osteoarthritis (OA) and rheumatoid arthritis (RA) remains unclear. This study aims to investigate the association between NPAR and the risk of OA and RA.

**Methods:**

This cross-sectional study analyzed data from 92,062 American adults in the NHANES database between 1999 and 2016. Various statistical analyses were conducted to investigate the associations between NPAR and the risks of OA and RA, including multivariable logistic regression, subgroup analysis, smooth curve fitting, and threshold effect analysis.

**Results:**

After screening, the final study population included 36,147 participants, with 3,881 individuals diagnosed with OA and 2,178 with RA. After adjusting for confounding factors, higher NPAR levels were associated with an increased risk of RA (OR=1.05; 95% CI: 1.03-1.07; *P* <0.0001), but not with OA (OR=1.01; 95% CI: 0.99-1.02; *P* =0.755). This association was remarkably consistent across subgroups by age, sex, body mass index (BMI), alcohol consumption, hypertension, diabetes, and smoking status. Further analyses using curve fitting and threshold effect models revealed a nonlinear association between NPAR and RA, with an inflection point identified at 15.56.

**Conclusion:**

High levels of NPAR is positively associated with the prevalence of RA. This provides us with new insights for the management and treatment of RA patients.

## 1 Introduction

Osteoarthritis (OA) and rheumatoid arthritis (RA) are the two most common types of arthritis in the United States, contributing significantly to disability. Reports indicate that approximately 22.7% of individuals in the United States are affected by arthritis (1–3). OA primarily degrades the articular cartilage, leading to joint pain, stiffness, crepitus during movement, effusion, and reduced mobility. OA most commonly affects weight-bearing joints, particularly the knees and hips (4). A large-scale cohort study found that the prevalence of radiographically diagnosed knee OA was 11.4% in women and 6.8% in men (5). Risk factors for OA encompass age, gender, obesity, genetic predisposition, joint deformity, and injury (6). RA is an immune-mediated inflammatory disease that typically presents with pain, swelling, and stiffness in synovial joints. Women account for 75% of RA patients (7, 8). Without prompt treatment, RA can result in joint destruction, disability, and systemic complications (9, 10). In the United States, the annual direct medical costs associated with arthritis are estimated to be as high as $81 billion (11). Arthritis imposes a significant medical burden on both individuals and society. Consequently, early screening and identification of high-risk populations are crucial to facilitate timely interventions and enhance the effectiveness of arthritis management strategies (12–14).

Biomarkers play a crucial role in the assessment of arthritis. Traditional biomarkers such as Anti-citrullinated peptide/protein antibodies (ACPA) have demonstrated high specificity in identifying RA patients and have proven to be cost-effective (15, 16). In recent years, researchers have discovered several novel biomarkers that show considerable potential in arthritis patients. A study has shown that integrating plasma/serum biomarkers, such as citrullinated protein, hydroxyproline, and anti-cyclic citrullinated peptide, with a diagnostic algorithm can facilitate early diagnosis and subtyping of various arthritis forms (17). Despite their promise, the widespread implementation of these novel biomarkers in routine clinical practice remains limited due to challenges in accessibility and availability. Growing evidence suggests that chronic low-grade inflammation plays a pivotal role in the onset and progression of OA (18, 19). RA is primarily attributed to autoimmune responses, with CD4+ T lymphocytes, interleukin-6 (IL-6), and tumor necrosis factor-α (TNF-α) identified as key mediators in the initiation and perpetuation of inflammation (20). Studies have demonstrated that nutrition and dietary habits can modulate metabolic processes and immune responses in both OA and RA (21, 22). Considering the crucial roles of inflammation and nutrition in arthritis, identifying novel biomarkers based on these factors is essential for assessing disease risk in clinical settings and guiding targeted interventions.

Neutrophils, essential components of the innate immune system, serve as cost-effective and easily obtainable markers for assessing inflammatory processes. The critical role of neutrophils in the pathogenesis of OA and RA has been well documented (23, 24). Albumin, a protein with a molecular weight of 66-69 kDa, is essential in various physiological processes and serves as a crucial marker of nutritional status. The functions of albumin encompass the maintenance of plasma osmotic pressure, facilitation of transport, and antioxidant activity (25). The neutrophil percentage to albumin ratio (NPAR), a composite biomarker derived from neutrophil and albumin levels, has demonstrated efficacy in predicting inflammation in various conditions, such as acute kidney injury, septic shock, and rectal cancer (26). However, the association between NPAR and arthritis remains unexplored.

Therefore, this study primarily aimed to investigate the relationship between NPAR and OA and RA risk by performing a large population-based analysis of the comprehensive National Health and Nutrition Examination Survey (NHANES) database.

## 2 Methods

### 2.1 NHANES study population

**Figure 1:**
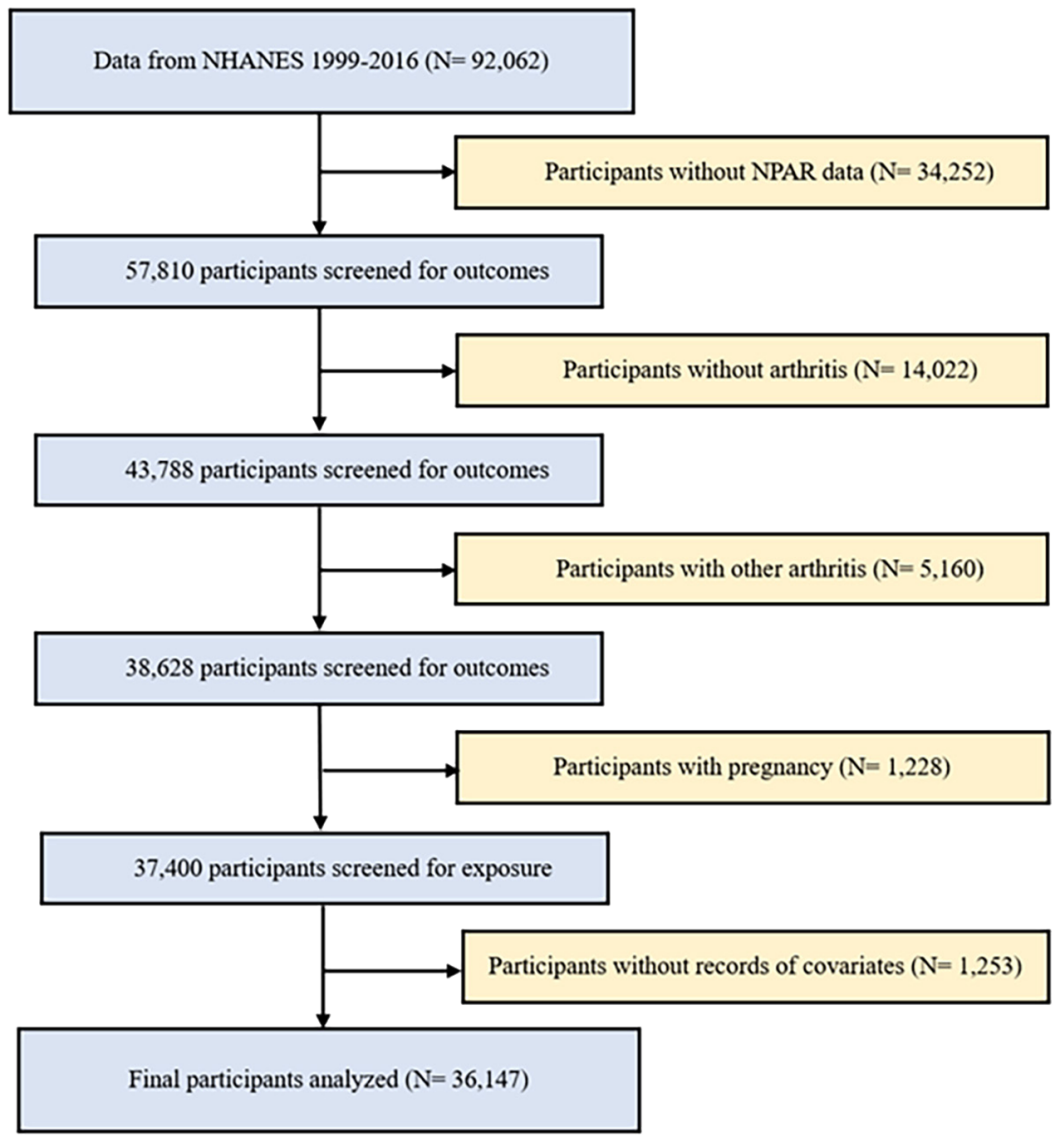
Flowchart of the participant selection from NHANES, 1999–2016.

This study utilized data from nine biennial cycles of the NHANES spanning from 1999 to 2016. All measurements and tests were meticulously carried out at on-site mobile testing facilities, guaranteeing adherence to the standardized procedures and protocols of data collection. Exclusion criteria were applied to study participants to ensure the authenticity and reliability of the results. This study employed the following exclusion criteria: (1) individuals with missing or incomplete data on arthritis and NPAR; (2) individuals diagnosed with other types of arthritis, as the control group consisted of individuals without any form of arthritis; (3) pregnant women, due to potential alterations in their metabolic profiles during pregnancy; and (4) participants with missing or unknown covariable data (**Figure 1**). After applying the exclusion criteria, a total of 36,147 participants were included in the study, comprising 3,881 patients with OA, 2,178 patients with RA, and 30,088 control participants without any form of arthritis.

### 2.2 Assessment of the NPAR and arthritis

The NPAR was calculated as: Neutrophil percentage (% of total WBC count) × 100/Albumin (g/dL) (27, 28).

Consistent with our previous study (29), the arthritis diagnosis of participants was confirmed using two self-reported questions. The first question enquired whether the participants had arthritis or not. Then, “Which type of arthritis?” Participants who responded with either “Osteoarthritis” or “Rheumatoid Arthritis” were classified as having OA or RA, respectively. A study found a strong correlation between self-reported arthritis and clinically diagnosed arthritis (30).

### 2.3 Assessment of covariables of interest

Numerous covariates were utilized to consider any confounding effects, including age; sex; five race categories; marital status; education level; body mass index (BMI, underweight: <18.5 kg/m2, normal weight: ≥18.5 kg/m2 and <25 kg/m2, overweight: ≥25 kg/m2 and <30 kg/m2, and obese: ≥30 kg/m2); and poverty-to-income ratio (PIR).

Participants who consumed more than 12 drinks in any given year were defined as drinkers. Participants were considered to have hypertension if the answer was “yes” on the self-report questionnaire for high blood pressure. Diabetes mellitus status was determined using the following criteria: self-reported diagnosis, glycosylated hemoglobin level of ≥6.5%, or fasting glucose level of ≥126 mg/dL. Participants were categorized as current, former and never smoker depending on whether they had smoked 100 or more cigarettes during their life and current smoking status.

Red blood cell count (RBC), hemoglobin (Hb), alanine aminotransferases (ALT), aspartate aminotransferase (AST) and Alkaline phosphatase (ALP) were measured from blood samples collected during the study visit.

### 2.4 Statistical analyses

Continuous variables were presented as mean± standard deviation (SD) and median and interquartile range (IQR), If a variable followed a normal distribution, a two-sample t-test was used for comparisons between groups; otherwise (if it did not follow a normal distribution), the rank sum test was employed. Categorical variables were presented as frequency and percentage, and Pearson’s chi-squared test was used to evaluate differences between groups. Multivariate logistic regression analysis was conducted to investigate the relationship between the NPAR and OA and RA. NPAR values were then categorized into quartiles, with the lowest quartile (Q1) serving as the reference group. Three models were constructed to assess the associations: Model 1, a crude model; Model 2, adjusted for age, sex, and race; and Model 3, further adjusted for marital status, education level, BMI, PIR, RBC, Hb, ALT, AST, alcohol consumption, hypertension, diabetes and smoking status. Subgroup analyses were conducted using multivariate logistic regression, adjusting for the confounding variables in Model 3. Interaction analyses was assessed using the likelihood ratio test to explore the heterogeneity of associations between different groups based on age, sex, BMI, alcohol consumption, hypertension, diabetes, and smoking status. To further investigate the association between NPAR and RA, we utilized a generalized additive model along with smooth curve fitting, which identified a non-linear relationship. Then we used a recursive technique to calculate the inflection point in the NPAR and RA. Finally, two-segment piecewise regression models were employed to perform a threshold effect analysis on both sides of the inflection point. All statistical analyses were conducted using R software (version 4.1.3) and EmpowerStats (version 6.0).

## 3 Results

### 3.1 Individual socio-demographic and health characteristics of the OA, RA and non-arthritis groups

The study participants had a mean age of 45.36 ± 17.07 years, with males accounting for 52.75% of the sample. 42.24% of the participants were non-Hispanic white, and more than half had education beyond high school. The self-reported prevalence of RA was 6.03%, and the self-reported prevalence of OA was 10.74%. OA and RA patients were older and more likely to be female compared to non-arthritis participants. Furthermore, significant differences were observed in race, marital status, education level, BMI, PIR, alcohol consumption, hypertension, status, smoking status, RBC, Hb, ALT, ALP between the non-arthritis, OA, and RA groups. Patients with OA and RA exhibited higher neutrophil percentages, lower albumin levels, and markedly elevated NPAR compared to individuals without arthritis (**Table 1**).

**Table 1:** Individual socio-demographic and health characteristics of the OA, RA and non-arthritis groups.

Characteristics	Non-arthritis (N = 30088)	OA (N = 3881)	P* value	RA (N = 2178)	*P^#^* value
	Mean ± SD Median (IQR) n (%)	Mean ± SD Median (IQR) n (%)		Mean ± SD Median (IQR) n (%)	
Age (years)			<0.001		<0.001
	45.36 ± 17.07	64.21 ± 13.46		60.93 ± 13.94	
	43.00 (31.00, 58.00)	66.00 (56.00, 75.00)		62.00 (51.25, 72.00)	
Sex			<0.001		<0.001
Male	15872 (52.8)	1409 (36.3)		896 (41.1)	
Female	14216 (47.3)	2472 (63.7)		1282 (58.9)	
Race			<0.001		<0.001
Mexican American	5896 (19.6)	340 (8.8)		364 (16.7)	
Other Hispanic	2601 (8.6)	227 (5.9)		171 (7.9)	
Non-Hispanic White	12708 (42.2)	2570 (66.2)		946 (43.4)	
Non-Hispanic Black	6042 (20.1)	540 (13.9)		606 (27.8)	
Other Races	2841 (9.4)	204 (5.3)		91 (4.2)	
Marital status			0.001		<0.001
Married or with partner	18547 (61.6)	2290 (59.0)		1190 (54.6)	
Single	11541 (38.4)	1591 (41.0)		988 (45.4)	
Education level			<0.001		<0.001
Less than high school	3388 (11.3)	369 (9.5)		401 (18.4)	
High school or GED	11169 (37.1)	1394 (35.9)		951 (43.7)	
Above high school	15531 (51.6)	2118 (54.6)		826 (37.9)	
BMI			<0.001		<0.001
Underweight	528 (1.8)	40 (1.0)		23 (1.1)	
Normal weight	9393 (31.2)	791 (20.4)		456 (20.9)	
Overweight	10465 (34.8)	1288 (33.2)		671 (30.8)	
Obese	9702 (32.3)	1762 (45.4)		1028 (47.2)	
PIR			<0.001		<0.001
≤1.3	8329 (27.7)	923 (23.8)		807 (37.1)	
(1.3, 3.5)	10299 (34.2)	1412 (36.4)		756 (34.7)	
≥3.5	9037 (30.0)	1270 (32.7)		444 (20.4)	
Unknown	2423 (8.1)	276 (7.1)		171 (7.9)	
Alcohol consumption			<0.001		<0.001
Yes	19976 (66.4)	2506 (64.6)		1308 (60.1)	
No	7566 (25.2)	1182 (30.5)		756 (34.7)	
Unknown	2546 (8.5)	193 (5.0)		114 (5.2)	
Hypertension			<0.001		<0.001
Yes	7976 (26.5)	2285 (58.9)		1286 (59.0)	
No	22112 (73.5)	1596 (41.1)		892 (41.0%)	
Diabetes			<0.001		<0.001
Yes	3677 (12.2)	909 (23.4)		633 (29.1)	
No	26411 (87.8)	2972 (76.6)		1545 (70.9)	
Smoking status			<0.001		<0.001
Current	6611 (22.0)	617 (15.9)		522 (24.0)	
Former	6498 (21.6)	1435 (37.0)		693 (31.8)	
Never	16979 (56.4)	1829 (47.1)		963 (44.2)	
RBC (million cells/uL)			<0.001		<0.001
	4.72 ± 0.50	4.53 ± 0.48		4.54 ± 0.51	
	4.72 (4.38, 5.06)	4.53 (4.20, 4.85)		4.55 (4.21, 4.87)	
Hb (g/dL)			<0.001		<0.001
	14.27 ± 1.53	13.88 ± 1.40		13.80 ± 1.55	
	14.30 (13.30, 15.40)	13.90 (13.00, 14.80)		13.80 (12.70, 14.90)	
ALT (U/L)			<0.001		<0.001
	26.26 ± 26.84	23.66 ± 14.83		24.61 ± 16.79	
	21.00 (16.00, 29.00)	20.00 (16.00, 26.00)		20.00 (16.00, 27.00)	
AST (U/L)			0.002		0.557
	25.86 ± 20.45	25.72 ± 13.38		26.39 ± 16.11	
	23.00 (19.00, 28.00)	23.00 (20.00, 28.00)		23.00 (19.00, 28.00)	
ALP (U/L)			<0.001		<0.001
	69.45 ± 24.46	72.64 ± 29.30		76.58 ± 28.43	
	66.00 (54.00, 81.00)	68.00 (56.00, 84.00)		72.00 (59.00, 88.00)	
Neutrophil percentage			<0.001		<0.001
	57.68 ± 9.32	59.15 ± 9.64		59.21 ± 10.06	
	58.20 (51.80, 63.90)	59.60 (53.50, 65.30)		59.30 (52.60, 65.90)	
Albumin(g/dL)			<0.001		<0.001
	4.30 ± 0.33	4.19 ± 0.32		4.15 ± 0.34	
	4.30 (4.10, 4.50)	4.20 (4.00, 4.40)		4.20 (3.90, 4.40)	
NPAR (dL/g)			<0.001		<0.001
	13.49 ± 2.47	14.20 ± 2.67		14.39 ± 2.90	
	13.45 (11.90, 15.05)	14.12 (12.48, 15.85)		14.24 (12.48, 16.08)	

Continuous variables were presented as mean± standard deviation (SD) and median and interquartile range (IQR).

Categorical variables were presented as frequency and percentage.

P* represents non-arthritis vs OA group; *P^#^* represents non-arthritis vs RA group.

### 3.2 Associations between the NPAR and RA and OA

**Table 2** illustrates the results of a multivariate logistic regression analysis examining the correlation between NPAR and self-reported RA. After adjusting for all covariates, the association between NPAR and self-reported RA remained statistically significant and positive (OR = 1.05; 95% CI: 1.03-1.07; *P* < 0.001). Moreover, individuals in the highest quartile (Q4) of NPAR exhibited a significantly increased risk of RA compared to those in the lowest quartile (Q1) as the reference group (OR = 1.25; 95% CI: 1.09-1.45; *P* = 0.002). **Table 3** displays that among OA patients, there were no significant associations observed between NPAR and the risk of OA (*P* = 0.755).

**Table 2:** Logistic regressions of RA by NPAR quantiles.

	Model 1^1^ Unadjusted OR, (95%CI), *P* value	Model 2^2^ Adjusted OR, (95%CI), *P* value	Model 3^3^ Adjusted OR, (95%CI), *P* value
	n= 32,266	n= 32,266	n= 27,368
RA			
NPAR	1.15 (1.13, 1.17) <0.001	1.09 (1.07, 1.11) <0.001	1.05 (1.03, 1.07) <0.001
Categories			
Quartile 1 (ref)	1.00	1.00	1.00
Quartile 2	1.09 (0.95, 1.25) 0.235	1.10 (0.96, 1.27) 0.175	0.05 (0.90, 1.22) 0.556
Quartile 3	1.30 (1.13, 1.48) 0.001	1.14 (1.00, 1.32) 0.058	1.02 (0.87, 1.18) 0.837
Quartile 4	2.06 (1.82, 2.33) <0.001	1.53 (1.34, 1.74) <0.001	1.25 (1.09, 1.45) 0.002
*P* for trend	<0.001	<0.001	0.002

OR, odds ratio; 95% Cl, 95% confidence interval.

^1^Model 1: No covariates were adjusted.

^2^Model 2: Adjusted for age, sex, and race.

^3^Model 3: Adjusted for age, sex, race, marital status, education level, BMI, PIR, RBC, Hb, ALT, AST, ALP, alcohol consumption, hypertension, diabetes and smoking status.

**Table 3:** Logistic regressions of OA by NPAR quantiles.

	Model 1^1^ Unadjusted OR, (95%CI), *P* value	Model 2^2^ Adjusted OR, (95%CI), *P* value	Model 3^3^ Adjusted OR, (95%CI), *P* value
	n= 33,969	n= 33,969	n= 28,897
OA			
NPAR	1.12 (1.10, 1.13) <0.001	1.03 (1.01, 1.04) 0.001	1.01 (0.99, 1.02) 0.755
Categories			
Quartile 1 (ref)	1.00	1.00	1.00
Quartile 2	1.18 (1.06, 1.31) 0.002	1.06 (0.94, 1.18) 0.348	1.03 (0.91, 1.16) 0.665
Quartile 3	1.45 (1.31, 1.61) <0.001	1.07 (0.96, 1.19) 0.246	0.97 (0.86, 1.09) 0.628
Quartile 4	1.98 (1.80, 2.18) <0.001	1.14 (1.03, 1.27) 0.013	0.97 (0.86, 1.09) 0.643
*P* for trend	<0.001	0.012	0.463

OR, odds ratio; 95% CI, 95% confidence interval.

^1^Model 1: No covariates were adjusted.

^2^Model 2: Adjusted for age, sex, and race.

^3^Model 3: Adjusted for age, sex, race, marital status, education level, BMI, PIR, RBC, Hb, ALT, AST, ALP, alcohol consumption, hypertension, diabetes and smoking status.

### 3.3 Subgroup analyses

**Figure 2:**
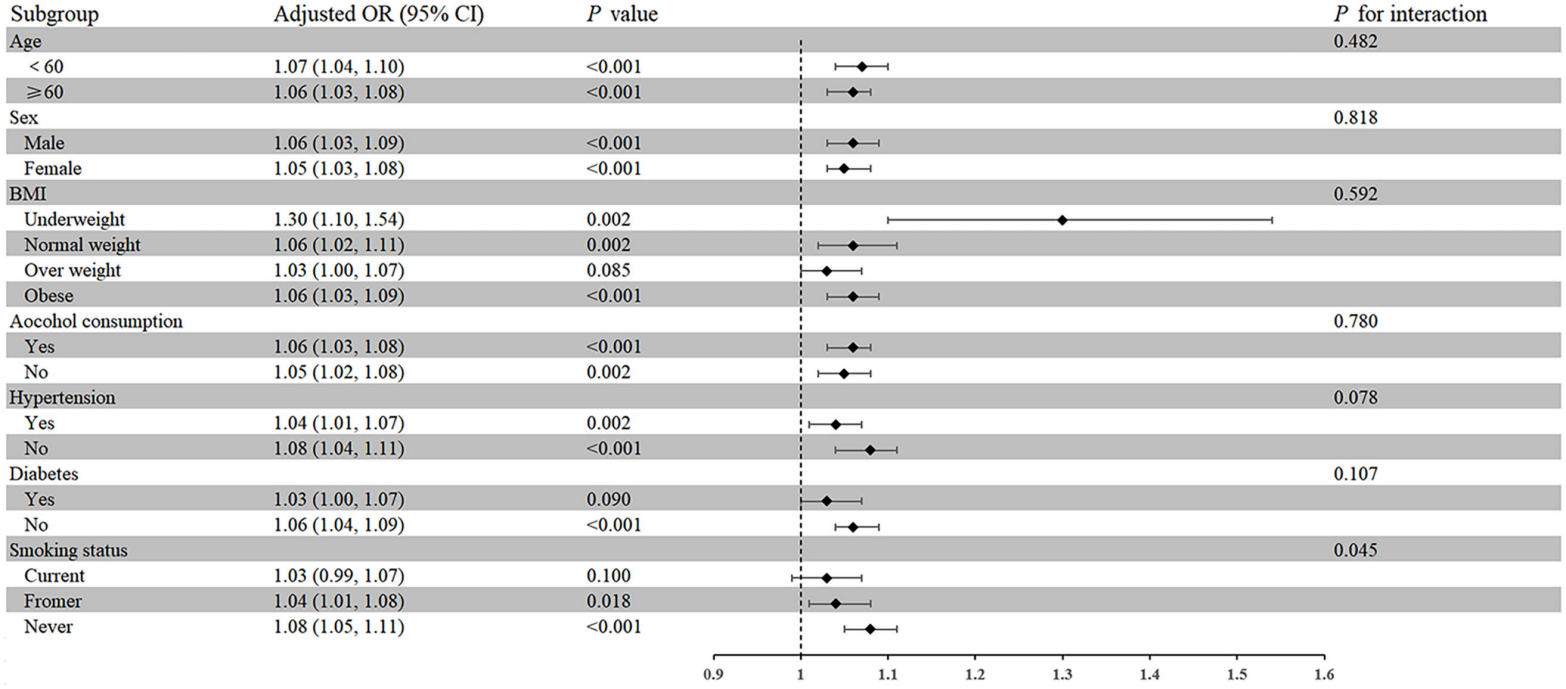
ORs of the subgroups for RA based on NPAR.

A subgroup analysis of seven factors (age, sex, BMI, alcohol consumption, hypertension, diabetes, and smoking status) was conducted to evaluate the strength and potential variations of the association between NPAR and self-reported RA among different populations (**Figure 2**). Moreover, except for subgroups with overweight, diabetes, and current smoking, the association between NPAR and RA was positive in all other subgroups (*P* < 0.05). Interaction tests showed that the association between NPAR and RA was more significant in never smokers than in current smokers and former smokers (OR = 1.08; 95% CI: 1.05-1.1, *P* for interaction < 0.05).

### 3.4 Nonlinear association between the NPAR and RA

**Figure 3:**
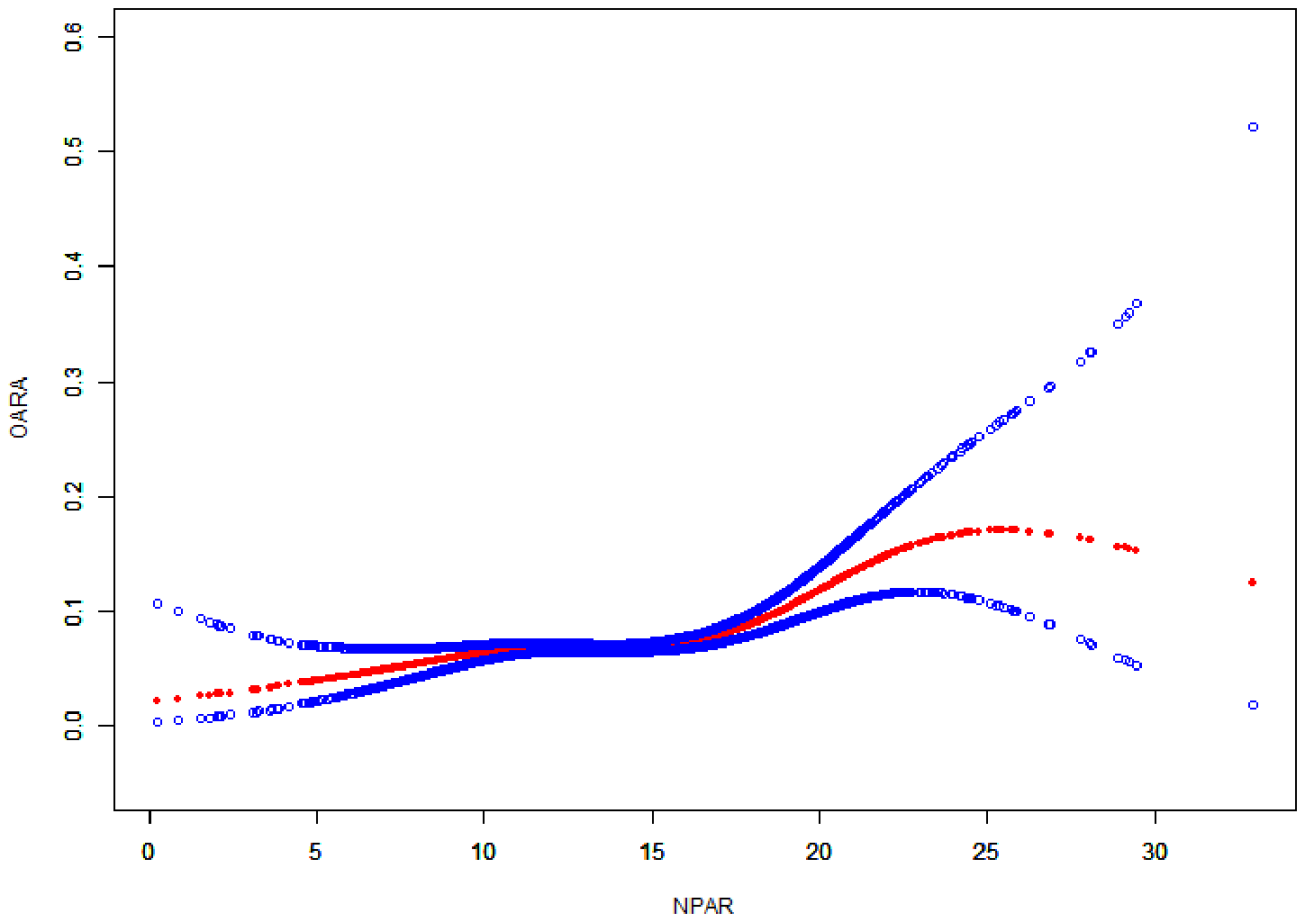
Smooth curve fitting analysis of the relationship between NPAR and RA.

The smoothing curve (**Figure 3**) revealed a nonlinear relationship between NPAR and self-reported RA. Through threshold effect analysis, we identified an inflection point at 15.56 dL/g (**Table 4**). When NPAR was below 15.56 dL/g, no statistically significant association was observed between NPAR and RA (OR = 1.02, 95% CI: 0.99-1.05; *P* = 0.207). However, when NPAR exceeded 15.56 dL/g, a significant positive correlation was found between NPAR and RA (OR = 1.12, 95% CI: 1.08-1.17; *P* < 0.001).

**Table 4:** Analysis of the threshold effect of NPAR and RA measured by the two-segment piecewise regression model.

RA	Adjusted OR (95% CI) *P* value
NPAR	
The standard linear mode	1.05 (1.03, 1.07) <0.001
Inflection point 1	15.56
NPAR < Inflection point1	1.02 (0.99, 1.05) 0.207
NPAR > Inflection point1	1.12 (1.08, 1.17) <0.001
Log-likelihood ratio1	0.001
Inflection point 2	19.94
NPAR < Inflection point2	1.17 (1.08, 1.26) <0.001
NPAR > Inflection point2	1.07 (0.95, 1.21) 0.239
Log-likelihood ratio2	0.310

## 4 Discussion

This study investigated the association between NPAR and the prevalence of self-reported OA and self-reported RA in a large sample of U.S. adults, including 3,881 OA patients, 2,178 RA patients, and 30,088 non-arthritis individuals. Multivariable logistic regression analysis revealed a positive association between NPAR and RA, which remained significant after adjusting for confounding factors. Additionally, subgroup analyses and interaction tests demonstrated that the association between NPAR and RA was consistent across various demographic and clinical subgroups, such as age, sex, BMI, alcohol consumption, hypertension, and diabetes. However, a significant interaction effect was observed for smoking status, where the association was stronger among never smokers compared to current and former smokers. Smooth curve fitting and threshold effect analysis revealed a nonlinear relationship between NPAR and RA, with an inflection point at 15.56 dL/g. In contrast, multivariable logistic regression analysis did not identify a statistically significant association between NPAR and OA. In conclusion, NPAR may be a useful tool for monitoring the prevalence of RA among U.S. adults, potentially aiding in risk stratification and early intervention strategies for susceptible populations.

In recent years, numerous studies have identified critical biomarkers in patients with arthritis. Zhao et al. found a significant inverse correlation between the systemic immune-inflammation index and serum Klotho levels, indicating that Klotho may have a protective role in the inflammatory response of RA patients and could be a potential therapeutic target for RA (3). Huang et al. demonstrated an inverse U-shaped nonlinear relationship between lipid accumulation product (LAP) level and OA, suggesting that LAP is a more reliable predictor of OA than traditional obesity measures like BMI, which could aid in OA prevention and treatment (31). Furthermore, using data from the NHANES database, Zhou et al. verified that an elevated neutrophil-to-lymphocyte ratio independently predicts a higher risk of all-cause and cardiovascular mortality in adults with RA, which could improve risk stratification and prognosis in RA patients (32). Wang et al. demonstrated that elevated NPAR levels were significantly associated with increased cardiovascular disease prevalence (33). Similarly, Li et al. found that NPAR exhibited a J-shaped association with all-cause mortality and a positive linear association with cardiovascular disease mortality among individuals with diabetes (34). These studies underscore the potential usefulness of novel serum biomarkers derived from routine blood tests in improving the diagnosis, risk stratification, and prognostic assessment of disease. However, the association between a novel inflammatory index, the NPAR, and arthritis has not been investigated to date.

These studies underscore the potential usefulness of novel serum biomarkers derived from routine blood tests in improving the diagnosis, risk stratification, and prognostic assessment of arthritis. However, the association between a novel inflammatory index, the NPAR, and arthritis has not been investigated to date.

Our study observed a significant positive association between elevated NPAR and the presence of self-reported RA, but no significant association with self-reported OA. NPAR can serve as an additional serum biomarker to complement the evaluation of RA. Recent studies have revealed associations between NPAR and various diseases, indicating its potential as a research target. For example, He et al. demonstrated a positive correlation between NPAR and the incidence of diabetic retinopathy, suggesting that NPAR could help assess the risk of retinopathy in diabetic patients and guide personalized treatment strategies (35). Similarly, Jiao et al. demonstrated that NPAR exhibited good sensitivity and specificity in assessing the one-year postoperative prognosis of elderly hip fracture patients, helping to identify individuals at high risk of post-operative mortality (36). Furthermore, a cohort study using NHANES data by Lan et al. found that that elevated NPAR was significantly associated with increased all-cause mortality, cardiovascular disease mortality, and mortality from chronic lower respiratory disease in patients with chronic obstructive pulmonary disease (COPD), suggesting that NPAR may be a better predictor of mortality than other hematological indices in COPD patients (37). In another study utilizing NHANES database, researchers found that NPAR exhibited excellent discriminatory ability for nonalcoholic fatty liver disease (NAFLD) in non-diabetic patients, with an area under the receiver operating characteristic curve of 0.810 (95% CI: 0.794–0.825), a sensitivity of 0.761, and a specificity of 0.715. Furthermore, the findings suggested that a higher NPAR is significantly associated with an increased risk of NAFLD, and may be a more effective biomarker for predicting NAFLD than albumin and neutrophil percentage alone (28). NPAR has unexpected potential in diagnosing, monitoring, and prognosticating various diseases. Given its accessibility and cost-effectiveness in routine medical practice, NPAR may provide additional benefits in the diagnosis and treatment of RA patients.

Elevated NPAR levels in RA patients may be attributed to increased neutrophil counts or decreased albumin levels. Excessive neutrophils release various proinflammatory mediators, such as reactive oxygen species, proteolytic enzymes, and cytokines (23). These mediators directly damage synovial tissue and stimulate the activation of other immune cells, including macrophages and T lymphocytes, which further amplifies the inflammatory response (38). In RA patients, the blood and synovial fluid show elevated levels of cytokines, such as granulocyte-macrophage colony-stimulating factor, TNF-α, IL-1β, and interferon. These cytokines delay neutrophil apoptosis through the upregulation of myeloid cell leukemia-1, phosphorylation of nuclear factor-kappa B, and increased caspase-9 activity, prolonging the lifespan of neutrophils and potentially increasing the absolute peripheral blood neutrophil count (39). Moreover, the increased tendency of neutrophils to form neutrophil extracellular traps in RA patients leads to the release of anti-citrullinated protein antibody and the induction of inflammatory cytokines, such as IL-6 and IL-8. These cytokines may then promote neutrophil production and survival, creating a vicious cycle that perpetuates the inflammatory process (40–42). Concurrently, the persistent inflammatory state may also impair the liver’s ability to synthesize albumin. Previous studies had demonstrated that inflammatory cytokines, such as IL-6 and TNF-α, can suppress hepatocyte albumin production, likely due to a reduction in albumin mRNA concentrations caused by decreased gene transcription, resulting in a decline in albumin synthesis (43). During the acute phase of inflammation, C-reactive protein (CRP) levels increase while albumin levels decrease, possibly due to the liver’s altered synthesis priorities (44, 45). The decrease in albumin levels in RA may also be related to other factors, such as inflammation-induced increased capillary permeability and increased protein loss due to RA-associated kidney involvement (44, 46, 47).

CRP and erythrocyte sedimentation Rate (ESR) are widely used and valuable indicators of systemic inflammation in RA and NPAR offers a unique perspective by virtue of its composite nature, integrating both inflammatory activity and nutritional status. The inclusion of albumin in the NPAR calculation provides valuable information regarding the patient’s nutritional status, a critical factor in RA. Chronic inflammation in RA patients often induces a catabolic state, leading to malnutrition and hypoalbuminemia, both of which have been associated with increased disease activity and poorer clinical outcomes (48, 49). While CRP primarily reflects the systemic acute-phase response and ESR can be influenced by factors beyond inflammation, NPAR may offer a more direct reflection of neutrophil-driven inflammation within the synovial joints, a central process in RA pathogenesis (42). Furthermore, NPAR’s reliance on readily available laboratory values, obtained from routine blood tests, renders it an accessible and cost-effective tool for routine clinical practice. Therefore, we propose that NPAR offers unique and complementary insights into the complex interplay of inflammation and nutritional status in RA, warranting further investigation as a potential biomarker for disease activity, prognosis, and treatment response.

These findings align with the well-established differences in the pathogenic mechanisms of RA and OA. While RA is characterized by systemic autoimmune-mediated inflammation, OA is primarily driven by mechanical joint injury, age-related degeneration, and localized inflammation (11, 50). These distinctions underscore the need for personalized diagnostic and therapeutic strategies for arthritis and further research to elucidate the complex mechanisms underlying these conditions.

This study provided robust evidence supporting a positive correlation between NPAR and RA, while demonstrating no association with OA, contributing to the arthritis literature. By leveraging the extensive sample size of the NHANES database, the findings are more comprehensive, representative, and generalizable. Furthermore, NPAR is inexpensive and easily accessible in clinical practice. When neutrophil percentage and albumin levels do not substantially deviate from normal ranges, NPAR may aid in identifying individuals with unrecognized RA risk.

However, this study has several limitations. First, the ascertainment of OA and RA diagnoses was based on self-reported data, without further confirmation from medical records, which is susceptible to recall and information bias. This cross-sectional design precludes the establishment of a temporal and causal relationship between NPAR and RA. While this study included numerous covariates to reduce the influence of confounding factors, it did not account for additional confounders that are more challenging to extract from the NHANES database, such as occupational exposure, genetic factors, and medication use. Moreover, a limitation of this study is the challenge in distinguishing between RA-specific processes and general inflammatory activity, which could potentially influence our findings. To address this, we have outlined several directions for future research, including prospective, real-world cohort studies, and investigations that account for or stratify by infections and other comorbidities. Furthermore, it is necessary to evaluate the performance of NPAR against established RA biomarkers, such as CRP, ESR, rheumatoid factor and ACPA. Finally, we emphasize the need for mechanistic studies, including *in vivo* investigations, to better understand the biological basis of our findings. These future studies are crucial for validating our findings and further elucidating the clinical utility of NPAR in RA.

## 5 Conclusion

In conclusion, this study reveals a positive correlation between NPAR and the risk of RA, while no association was observed with OA. The findings enhance our understanding of the roles of systemic inflammation and albumin levels in the pathogenesis of RA, potentially offering valuable insights for early detection, prevention, and targeted therapeutic approaches for RA management. Nevertheless, future research with larger sample sizes and longitudinal study designs is warranted to validate these findings.

## Data Availability

Publicly available datasets were analyzed in this study. This data can be found here: https://www.cdc.gov/nchs/nhanes/.
